# Quantitative Analysis of Colostrum Bacteriology on British Dairy Farms

**DOI:** 10.3389/fvets.2020.601227

**Published:** 2020-12-07

**Authors:** Robert M. Hyde, Martin J. Green, Chris Hudson, Peter M. Down

**Affiliations:** School of Veterinary Medicine and Science, University of Nottingham, Leicestershire, United Kingdom

**Keywords:** cattle, dairy, colostrum, bacteriology, bootstrap

## Abstract

Total bacterial counts (TBC) and coliform counts (CC) were estimated for 328 colostrum samples from 56 British dairy farms. Samples collected directly from cows' teats had lower mean TBC (32,079) and CC (21) than those collected from both colostrum collection buckets (TBC: 327,879, CC: 13,294) and feeding equipment (TBC: 439,438, CC: 17,859). Mixed effects models were built using an automated backwards stepwise process in conjunction with repeated bootstrap sampling to provide robust estimates of both effect size and 95% bootstrap confidence intervals (BCI) as well as an estimate of the reproducibility of a variable effect within a target population (stability). Colostrum collected using parlor (2.06 log cfu/ml, 95% BCI: 0.35–3.71) or robot (3.38 log cfu/ml, 95% BCI: 1.29–5.80) milking systems, and samples collected from feeding equipment (2.36 log cfu/ml, 95% BCI: 0.77–5.45) were associated with higher TBC than those collected from the teat, suggesting interventions to reduce bacterial contamination should focus on the hygiene of collection and feeding equipment. The use of hot water to clean feeding equipment (−2.54 log cfu/ml, 95% BCI: −3.76 to −1.74) was associated with reductions in TBC, and the use of peracetic acid (−2.04 log cfu/ml, 95% BCI: −3.49 to −0.56) or hypochlorite (−1.60 log cfu/ml, 95% BCI: −3.01 to 0.27) to clean collection equipment was associated with reductions in TBC compared with water. Cleaning collection equipment less frequently than every use (1.75 log cfu/ml, 95% BCI: 1.30–2.49) was associated with increased TBC, the use of pre-milking teat disinfection prior to colostrum collection (−1.85 log cfu/ml, 95% BCI: −3.39 to 2.23) and the pasteurization of colostrum (−3.79 log cfu/ml, 95% BCI: −5.87 to −2.93) were associated with reduced TBC. Colostrum collection protocols should include the cleaning of colostrum collection and feeding equipment after every use with hot water as opposed to cold water, and hypochlorite or peracetic acid as opposed to water or parlor wash. Cows' teats should be prepared with a pre-milking teat disinfectant and wiped with a clean, dry paper towel prior to colostrum collection, and colostrum should be pasteurized where possible.

## Introduction

Bovine neonates are born agammaglobulinemic (1) and consequently must acquire immunity via the ingestion of appropriate quantities of high quality colostrum within the first few hours of life (2). A failure of passive immunity transfer (FPT) in dairy calves has been associated with an increased risk of preweaning morbidity and mortality (3, 4), as well as longer term effects such as increased age at first calving and reduced milk production (5). A recent meta-analysis concluded that calves experiencing FPT are 1.5 times more likely to be treated for diarrhea, 1.8 times as likely to be treated for respiratory disease and two times as likely to die (6). Despite the clear negative implications, recent research suggests FPT is common in UK dairy calves, with around 26% experiencing FPT as measured by total protein (TP) < 5.6 g/dl (7).

Failure of passive immunity transfer has been associated with suboptimal colostrum feeding volume, timing, quality and also microbiological hygiene (2). Microbiological contamination of colostrum can not only represent a significant risk for FPT through reduced efficiency of immunoglobulin absorption (8), but also act as a vehicle for the transmission of pathogenic organisms to the neonatal calf (9). Higher bacterial levels have been hypothesized to reduce immunoglobulin (Ig) absorption by the binding and neutralizing of Ig by bacteria, pathogenic bacteria damaging intestinal epithelial cells and reducing permeability to Ig, and nonspecific pinocytosis of bacteria blocking the absorption of Ig molecules (10). In addition to effects on FPT and disease early in life, colostrum hygiene has also been identified as a critical control point in the prevention of paratuberculosis (11).

Given the importance of colostrum bacterial levels, several researchers have attempted to provide benchmarking estimates of contamination levels at a national level. Previous thresholds for classification of bacterial contamination have been suggested at >100,000 colony-forming units (cfu) per ml and >10,000 cfu/ml for total bacterial counts (TBC) and coliform counts (CC), respectively (12). North American studies have reported 36% of samples exceeding TBC thresholds in Canada (13), and 85% in the US (12), with later studies of 67 farms in 12 states finding 43 and 17% samples exceeding TBC and CC thresholds, respectively (14). Only 18% of 255 samples from 44 Columbian herds were found to fail on TBC (15). Of 268 NZ samples taken of pooled colostrum, 91 and 91% failed for TBC and CC, respectively (16). Of 221 Australian colostrum samples, 42 and 28% of samples failed on TBC and CC, respectively, with only 20% meeting both standards for immunoglobulin (>50 g/L) and microbiological quality (17), reinforcing previous Australian studies which found 42 and 6% of samples exceeded TBC and CC, respectively, with only 23% meeting all standards for TBC, CC and immunoglobulin levels (18). Previous studies examining colostrum bacteriological levels in Irish dairy herds reported 57% of 214 samples exceeding TBC and 33% exceeding CC thresholds, with significant variation between farms (9), however, there have been no published studies on colostrum hygiene levels on GB dairy farms to date. In addition to a paucity of information around current GB colostrum bacterial levels, there is also a lack of knowledge around specific factors that may influence the bacterial contamination of colostrum (18).

The source of bacteria present in colostrum includes the mammary gland itself as well as contamination or proliferation during harvesting, storage or feeding (12, 13, 19). Previous research has shown that colostrum contamination is generally extremely low or zero when collected directly from the gland (mean log TBC: 1.44 cfu/ml), with significant bacterial contamination occurring during the harvest process (mean log TBC: 4.99 cfu/ml), suggesting steps to prevent colostrum contamination should focus largely on collection methods (19). Storage method also has an effect on bacterial levels, with colostrum stored at warmer conditions (22 degrees C) having >42 times more bacteria present and resulting in a serum IgG concentration almost twice as low compared with colostrum either pasteurized, untreated or stored at 4°C for 2 d (20), and bacterial levels have been shown to be significantly reduced when freeze-thawing colostrum (21). Irrespective of the source of colostrum contamination, it has been found that heat treatment is associated with reduced bacterial levels, improved health status and decreased mortality, even when receiving appropriate colostrum volume (22). This reinforces previous findings that calves fed heat-treated colostrum have significantly higher serum IgG concentrations, and a significantly lower risk of diarrhea than those fed fresh colostrum (10), suggesting colostrum pasteurization may be an effective method of reducing colostrum contamination levels. Whilst colostrum pasteurization is likely to be effective at reducing colostrum bacterial levels, it is unknown how many GB farms currently pasteurize colostrum.

Whilst there are many farm level factors associated with colostrum contamination levels, it is likely that protocols aiming to prevent colostrum contamination are likely to vary between farms (9), and it is important that veterinary advisors are able to recommend interventions that are relevant to the majority of farms. The relative importance of management factors is essential for optimal decision making on-farm (23). Bootstrapping allows for the estimation of robust coefficients (24) and estimates of variable stability: an estimate of the reproducibility of a variable effect within a target population. The use of bootstrapping alongside regression techniques has been utilized in studies identifying a relatively small number of variables having a large and consistent effect on animal health outcomes (23), and the identification of these variables can provide a succinct number of practical recommendations for veterinarians.

This study aims to provide a current benchmark of colostrum bacterial levels, investigate factors associated with increased levels of bacterial contamination of colostrum on GB dairy farms and provide practical recommendations for a small number of factors found to have the largest effect on colostrum hygiene on the largest number of farms.

## Materials and Methods

### Farm Selection

Dairy farms were selected at random from a list of suppliers to a large supermarket consisting of dairy farms in England, Scotland and Wales. Randomization was performed using the sample_n() function from the tidyverse package (25) in R statistical software (26). 120 farms were initially selected and were sent an initial information letter, followed up with a telephone call to recruit farms until 60 were recruited as part of a wider calf research project. Recruited farms were visited by one researcher between 17^th^ December 2018 - 14^th^ February 2019 and provided with a colostrum collection kit (Quality Milk Management Services, Wells UK). One farmer on each farm was trained by the researcher on the collection and posting of colostrum samples. To replace farms leaving the wider calf research project an additional 31 farms were enrolled by the same methodology and were visited by the same researcher between 6^th^ December 2019 and 29^th^ January 2020 and were also provided with colostrum collection kits and training on collection protocols.

### Colostrum Collection Protocol

Farmers were encouraged to take six colostrum samples from as close to the calf feeding point as possible, for example farms bottle-feeding colostrum to all calves should take samples from the bottle teat. Where colostrum feeding involved multiple sources, farmers were asked to collect samples from all sources, for example farms where calves would suckle colostrum from the dam in most cases and receive supplementary colostrum by esophageal tube feeding when necessary should take some samples from the cows' teat, and some from the esophageal tube. Farmers were asked to freeze samples as soon as possible after collection, with all six samples being collected within 1 month of each other. Sample pots contained glycerol as a cryopreservative. After collecting 6 samples, farmers were asked to place the samples in a pre-packaged insulated box containing ice packs provided to the farmer. These boxes were couriered to the laboratory (Quality Milk Management Services, Wells UK) for analysis.

### Microbiological Analysis

Samples were analyzed using standard laboratory methods for milk (27) as previously described (28). Ten microlitres of secretion was inoculated onto sheep blood agar and Edwards agar and 100 μl was inoculated onto MacConkey agar. Total bacterial count and coliform counts were estimated after incubation for 72 h at 30 degrees C and 37 degrees C, respectively.

### Colostrum Collection Questionnaire

A submission form was submitted alongside colostrum samples including the cow id, date of sampling and sample collection location. Upon receiving the colostrum samples, farmers were contacted via telephone and asked about colostrum collection and equipment cleaning protocols used to collect the colostrum samples. Where samples were taken directly from the cows' teat, the method used to clean collection buckets or feeding equipment was deemed irrelevant, and a “Not applicable” factor level was created. Similarly, where samples collected from the collection bucket but before using any feeding equipment, the methods used to clean feeding equipment was deemed irrelevant and a “Not applicable” factor level was created. The percentage of calves receiving manually fed colostrum feeds (feeds by bottle or tube as opposed to suckling from the dam) and the volume of first feeds was also recorded.

### Descriptive Analysis

All data analysis was conducted in R (26). The percentage of colostrum samples failing in terms of TBC and CC was calculated for each farm, with a “failure” being when >100,000 for TBC and >10,000 for CC as previously suggested by McGuirk and Collins (12).

### Statistical Analysis

Both TBC and coliform counts were natural log transformed after the addition of 1 to all counts. Samples with missing data were removed from the dataset. Continuous variables were scaled (divided by one standard deviation) and centered prior to modeling using the preProcess function within the caret package (29). Categorical variables for cleaning frequency were releveled to include as few relevant categories as possible; when cleaning frequency was recorded as “Less than daily” or “Daily” these were recategorised to “Less than each use”.

For model building, a bootstrap sample was taken from the dataset (sampling with replacement to create a sample of the same size as the original dataset). A mixed model was created from the bootstrapped data sample using the lmer function from the lmerTest package (30) with log TBC or log CC as model outcomes in respective models. Farm was included as a random effect, and all other colostrum management variables were included as fixed effects as shown in Table 1. The following model equation was used for the mixed model:

$$\begin{array}{l} Y_{ij}=\;\mu +\beta_{1}X_{1ij}+\;\beta_{2}X_{2ij}\ldots +\;U_{j}+\;\epsilon \end{array}$$

Where *Y*_*ij*_ is the log TBC or CC of the *i*th sample on the *j*th farm. *X*_1*ij*_ represents covariates at the sample farm level, with corresponding coefficients represented by β_1_, and *X*_2*ij*_ representing covariates at the sample farm level, with corresponding coefficients represented by β_2_. μ represents the intercept, β represents explanatory variables, *U*_*j*_ as the farm specific random effect for the *j*th farm, and ϵ as the random error. The assumed distributions of *U* and ϵ are normal, with mean zero and variance θ *U* and θ ϵ respectively.

**Table 1:** Colostrum management variables and factor levels available as fixed effects for model building.

**Variable**	**Levels (% of samples)**
Sample collection point	Cows teat (17.8%), Colostrum collection bucket (36.3%), Feeding teat (24.4%), Esophageal tube (21.6%)
Number of days between calving pen clean out	Numeric
Pre-milking teat disinfection used	No (17.4%), Yes (82.6%)
Teat dry wiped prior to colostrum collection	No (17.7%), Yes (82.3%)
Milking system	Parlor (67.0%), Robot (15.2%), Not applicable (17.8%)
Frequency of colostrum collection equipment cleaning	Each use (21.0%), Less than each use (61.2%), Not applicable (17.8%)
Method of colostrum collection equipment cleaning	Water (24.1%), Hypochlorite (16.5%), Parlor wash (28.7%), Peracetic acid (9.5%), Soap (3.7%), Not applicable (17.8%)
Hot water used to clean collection equipment	No (38.1%), Yes (44.2%), Not applicable (17.8%)
Frequency of colostrum feeding equipment cleaning	Each calf (31.1%), Less than each calf (14.9%), Not applicable (54.0%)
Method of colostrum feeding equipment cleaning	Water (10.4%), Hypochlorite (13.7%), Parlor wash (7.3%), Peracetic acid (7.3%), Soap (7.3%), Not applicable (54.0%)
Hot water used to clean feeding equipment	No (14.9%), Yes (31.1%), Not applicable (54.0%)
Colostrum frozen prior to sample collection	No (78.0%), Yes (4.3%), Not applicable (17.8%)
Colostrum pasteuriser used	No (78.9%), Yes (3.4%), Not applicable (17.8%)

An automated backwards stepwise selection process based on Akaike information criterion was used using the step function from the lmerTest package (30) to create a final mixed effects model for a given bootstrap sample, and variables from the final model were recorded alongside coefficient values. This process was then repeated 1,000 times, recording the presence of variables and their corresponding effect size in each iteration. Residuals were checked to ensure near normal distribution after building an automated backwards stepwise mixed effects model on the full dataset (i.e., without bootstrap sampling). Cross-validation (10-fold, repeated 10 times) was use for the full model and both internal and cross-validated R2 and MAE were assessed to ensure the model was not overfit. Interactions between significant predictors in the full model were checked and were included if *p* < 0.05.

Variable stability was calculated as the percentage of bootstrap models in which a given variable was selected. Mean coefficient values and 95% bootstrap confidence intervals (BCI) were calculated from coefficient values across all bootstrap samples in which a variable was selected. An estimate of significance as a “bootstrap *p*-value” was calculated as one minus the proportion of coefficient estimates on the majority side of zero (proportion below zero if the mean coefficient was above zero, and proportion above zero if mean coefficient was above zero). Variables with a bootstrap stability >10% and a bootstrap *p*-value < 0.025 were deemed to be both relatively stable and have reasonable effect size.

## Results

### Descriptive Analysis

A total of 356 samples were returned from 59 farms. Fifteen samples from six farms were either missing or damaged in transit and were removed from the dataset. Thirteen samples from three farms were removed due to missing or incomplete data on sample collection. Thirty-two farms failed to return any samples, with 19 farms failing to return samples from the first round of data collection, and 13 farms failing to return samples during the second round of data collection. The final dataset consisted of 328 samples from 56 farms. One hundred and fifty-one samples were collected from feeding equipment, with 80 (53.0%) being collected through a feeding teat and 71 (47.0%) being collected through an esophageal feeding tube. One hundred and nineteen samples were collected from a collection bucket and 58 samples were collected directly from the cow's teat. Pre-milking teat disinfection was used prior to colostrum collection for 271 (82.6%) samples compared with 57 (17.4%) samples where no pre-milking disinfection was used. Dry wiping of teats prior to colostrum collection was conducted for 270 (82.3%) samples compared with 58 (17.7%) with no dry wiping of teats. The frequency of the cleaning out of calving pens was between 3.5 and 90 d, with a mean and median of 27.9 and 28 d, respectively. Farmers reported manually feeding colostrum (by bottle or tube as opposed to suckling from the dam) to between 0 and 100% of calves, with a mean and median of 79.2 and 100%, respectively. Colostrum volume fed by farmers at first feed ranged from 2 to 5 L at first feed, with a mean and median of 3.1 and 3.0 L, respectively.

Of the 270 samples collected using milking equipment (i.e., excluding the 58 samples collected directly from the teat), 220 (81.5%) were collected through a milking parlor, and 50 (18.5%) through a robotic milking unit. Only 69 samples (25.6%) were submitted from farms where collection equipment (i.e., collection bucket) was cleaned after each use, with 201 (74.4%) being collected from farms where collection equipment was cleaned less frequently than after each use. Seventy-nine samples (29.2%) were collected from farms using water to clean collection equipment, 94 (34.8%) using parlor washings, 54 (20.0%) from farms using hypochlorite, 31 (11.5%) using peracetic acid, and 12 (4.4%) using soap. One hundred and forty-five samples (53.7%) were collected from farms using hot water to clean collection equipment, compared with 125 (46.3%) from farms that did not use hot water. Farms that used a colostrum pasteuriser accounted for 11 samples (4.1%), compared with 259 samples (95.9%) where a pasteuriser was not used. Colostrum was frozen prior to sample collection for 14 samples (5.2%) compared with 256 samples (94.8%) which were collected without prior freezing.

Of the 151 samples collected directly from feeding equipment (i.e., excluding the 58 samples collected directly from the teat and the 119 samples collected from the collection bucket) 102 samples (67.5%) were collected from farms where feeding equipment was cleaned every time it was used, and 49 samples (32.4%) when feeding equipment was cleaned less than each use. Thirty-four samples (22.5%) were collected from farms using water alone to clean feeding equipment, 24 (15.9%) using parlor washings, 45 (29.8%) from farms using hypochlorite, 24 (15.9%) using peracetic acid, and 24 (15.9%) from farms using soap. One hundred and two samples (67.5%) were from farms that used hot water to clean feeding equipment, compared with 49 (32.4%) from farms that did not use hot water.

Mean TBC and CC were 326,931 and 13,034 cfu/ml with median values of 14,800 and 1 cfu/ml, respectively. Ninety-seven (29.6%) samples had TBC results above threshold (>100,000 cfu/ml) and 25 samples (7.6%) had coliform counts above threshold (>10,000 cfu/ml).

Mean and median TBC were lower when samples were collected directly from the cow's teat, at 32,079 and 535, respectively, with only 6.9% of samples being above threshold, compared with collection from a collection bucket (mean 327,879, median 44,000, 35.3% above threshold) or feeding equipment (mean 439,438, median 18,100, 33.8% above threshold). Coliform counts were also lower when samples were collected from the cow's teat (mean 21, median 0, 0.0% above threshold) compared with samples taken from the collection bucket (mean 13,294, median 2, 7.6% above threshold) or feeding equipment (mean 17,859, median 1, 10.6% above threshold). A higher number of samples collected directly from the cow's teat had zero coliforms present, with 43 samples (74.1%) having zero coliforms when collected directly from the cow's teat compared with 48 samples (40.3%) taken from the collection bucket and 62 samples (41.0%) when collected from feeding equipment. In contrast, only one sample collected directly from a cows' teat, and zero samples collected from collection or feeding equipment had a zero TBC. A lower proportion of samples collected from cows' teats (6.9%) were above threshold for either TBC or CC than those collected from collection buckets (37.0%) or feeding equipment (34.4%).

### Statistical Analysis

No interactions were detected between significant predictors in the full (non-bootstrapped) model, and analysis of cross-validated and internal R2 and MAE suggested the model was not overfit. Predictor variables were assessed to evaluate correlations; since all correlations were <0.36 and cross validation provided no indication of over fitting, the full model was deemed to provide safe parameter estimates.

Thirteen variables were available for predicting both colostrum TBC and CC. After model building using automated backwards stepwise regression and bootstrap resampling, final models resulting in eight and seven variables being selected for TBC and CC respectively.

### Total Bacterial Counts

The use of a milking machine was associated with an increase in TBC compared with those collected directly from the cows' teat with a stability of 92.5% being associated with a 2.06 log cfu/ml (95% BCI: 0.35–3.71) increase when collected through a parlor, and a 3.38 log cfu/ml (95% BCI: 1.29–5.80) increase when collected through a robot. Sample collection point was also associated with increased TBC, with a stability of 87.3%, being associated with a 2.36 log cfu/ml (95% BCI: 0.77–5.45) increase when collected from feeding equipment compared with samples collected directly from cows' teats.

The use of hot water to clean feeding equipment was associated with reduced TBC with a stability of 85.9%, being associated with a −2.54 log cfu/ml (95% BCI: −3.76 to −1.74) reduction when hot water was used. The method of cleaning colostrum collection buckets was associated with TBC with a stability of 29.1%. Compared with water, cleaning colostrum collection buckets with peracetic acid was associated with a −2.04 log cfu/ml (95% BCI: −3.49 to −0.56) reduction in TBC, and a tendency toward reduced TBC was identified when cleaning with hypochlorite (−1.60 log cfu/ml, 95% BCI: −3.01 to 0.27) and soap (−1.14 log cfu/ml, 95% BCI: −3.01 to 0.27). No difference was detected when cleaning with parlor wash (0.47 log/cfu/ml, 95% BCI: −0.76 to 1.89) compared with water. The frequency of colostrum collection equipment cleaning was associated with TBC with a stability of 22.1%, and a 1.75 log cfu/ml (95% BCI: 1.30–2.49) increase when collection equipment was cleaned less than every time it was used. The wiping of teats prior to colostrum collection was associated with a reduction in TBC with a stability of 23.3% and a −1.97 log cfu/ml (95% BCI: −2.85 to −1.45) reduction in TBC. The use of a colostrum pasteuriser was associated with TBC with a stability of 10.9% and a −3.79 log cfu/ml (95% BCI: −5.87 to −2.93) reduction in TBC.

**Figure 1:**
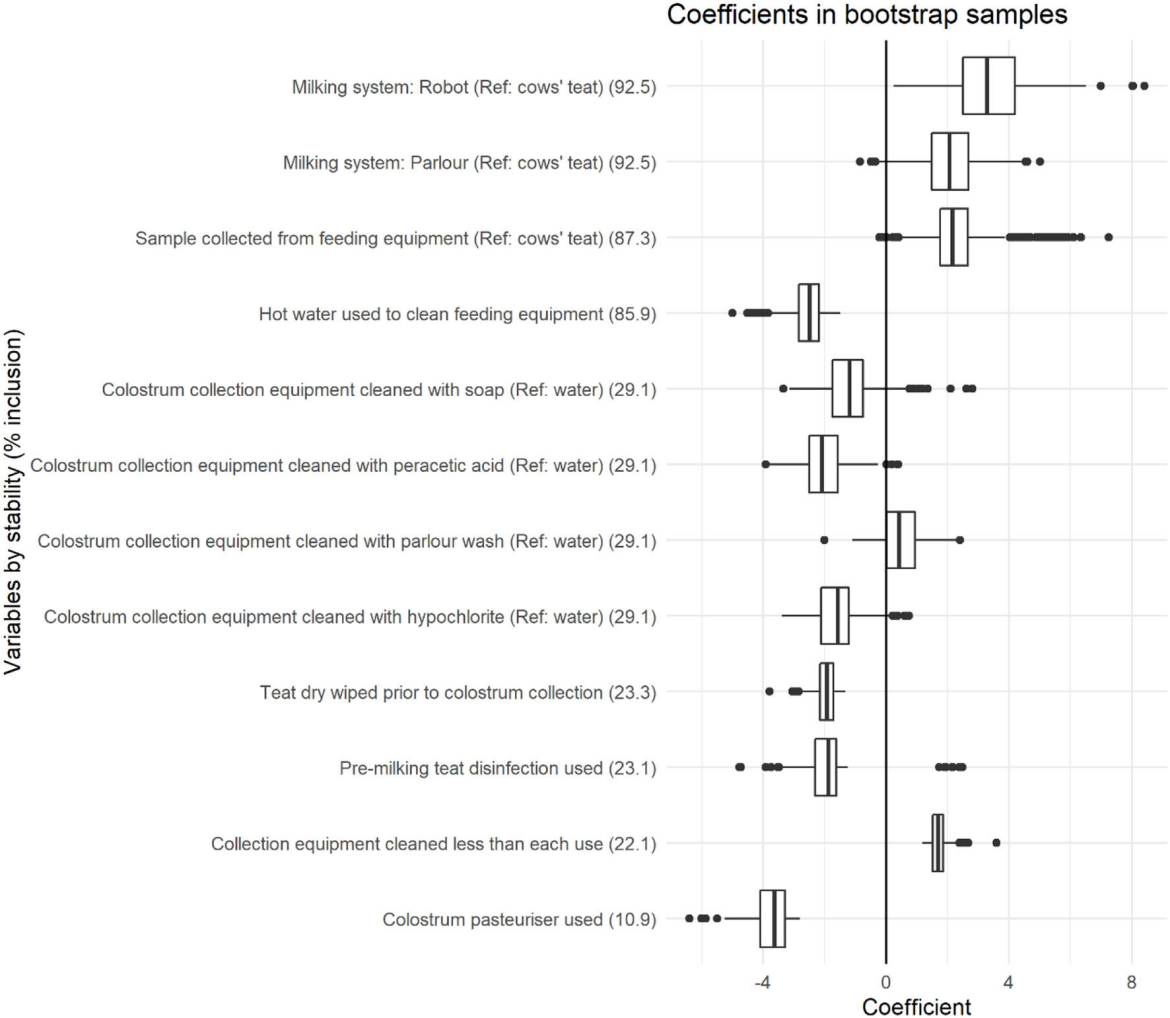
Coefficient distributions and variable stability for variables selected in at least 10% of models across 1,000 bootstrapped samples. Coefficient estimates represent the change in total bacterial count (log cfu/ml), and variable stability is presented within brackets for each variable.

**Figure 2:**
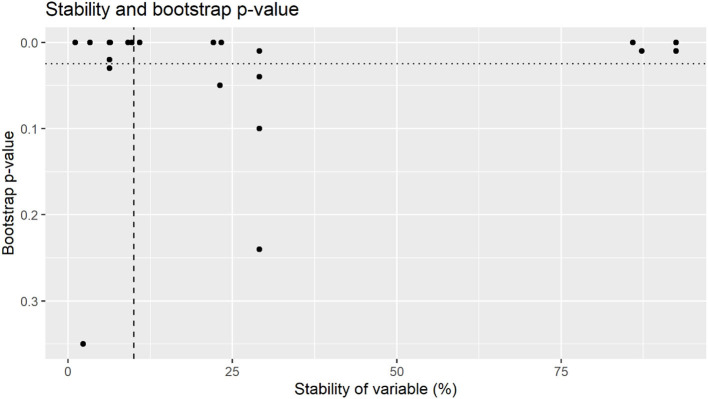
Bootstrap *p*-value by stability of variables for total bacterial counts. Variables were selected for final model were above 10% stability (dashed line) with a bootstrap *p*-value of <0.025 (dotted line).

Variables with >10% stability and <0.025 bootstrap *p*-value are depicted in Figure 1, with stability estimates of variables being presented in Figure 2. Coefficients, 95% BCI and stability estimates for all model variables are presented in Table 2.

**Table 2:** Stability, mean coefficient (log cfu/ml), 95% bootstrap confidence intervals and bootstrap *p*-value for all variables associated with total bacterial count.

**Variable**	**N**	**Stability (%)**	**Mean coefficient**	**95% bootstrap confidence interval**	**Bootstrap *P*-value**
Milking system: parlor (ref: cows' teat)	220	92.5	2.06	(0.35 to 3.71)	0.01
Milking system: robot (ref: cows' teat)	50	92.5	3.38	(1.29 to 5.80)	<0.01
Sample collected from feeding equipment (ref: cows' teat, *n* = 58)	151	87.3	2.36	(0.77 to 5.45)	0.01
Hot water used to clean feeding equipment	102	85.9	−2.54	(−3.76 to −1.74)	<0.01
Colostrum collection equipment cleaned with hypochlorite (ref: water)	54	29.1	−1.60	(−3.01 to 0.27)	0.04
Colostrum collection equipment cleaned with parlor wash (ref: water)	94	29.1	0.47	(−0.76 to 1.89)	0.24
Colostrum collection equipment cleaned with peracetic acid (ref: water)	31	29.1	−2.04	(−3.49 to −0.56)	0.01
Colostrum collection equipment cleaned with soap (Ref: water)	12	29.1	−1.14	(−2.55 to 1.13)	0.10
Teat dry wiped prior to colostrum collection	270	23.3	−1.97	(−2.85 to −1.45)	<0.01
Pre-milking teat disinfection used	271	23.1	−1.85	(−3.39 to 2.23)	0.05
Collection equipment cleaned less than each use	201	22.1	1.75	(1.30 to 2.49)	<0.01
Colostrum pasteuriser used	11	10.9	−3.79	(−5.87 to −2.93)	<0.01
Feeding equipment cleaned less than each calf	49	9.7	−2.13	(−2.98 to −1.63)	<0.01
Hot water used to clean collection equipment	145	9.1	−1.60	(−2.17 to −1.16)	<0.01
Sample collected from collection equipment (ref: cows' teat)	119	6.4	3.51	(2.36 to 4.38)	<0.01
Colostrum feeding equipment cleaned with hypochlorite (ref: water)	45	6.3	3.23	(0.25 to 5.29)	0.03
Colostrum feeding equipment cleaned with parlor wash (ref: water)	24	6.3	2.66	(0.96 to 4.47)	<0.01
Colostrum feeding equipment cleaned with peracetic acid (ref: water)	24	6.3	3.84	(1.34 to 5.75)	0.02
Colostrum feeding equipment cleaned with soap (ref: water)	24	6.3	2.29	(0.09 to 3.83)	0.03
Number of days between calving pen clean out	328	3.3	0.65	(0.54 to 0.84)	<0.01
Colostrum frozen prior to sample collection	14	2.3	0.75	(−2.53 to 3.34)	0.35
Colostrum collection equipment cleaning: not applicable	58	1.1	−4.03	(−4.64 to −3.48)	<0.01

N represents the number of samples where variable was “positive.”

### Coliform Counts

The use of hot water to clean both feeding equipment and collection equipment was associated with CC, with stabilities of 51.9 and 32%, respectively. The use of hot water to clean feeding equipment and collection equipment was associated with a −2.72 log cfu/ml (95% BCI: −4.01 to −1.82) and −1.72 log cfu/ml (95% BCI: −2.35 to −1.26) reduction in CC, respectively. The sample collection point was associated with CC with stabilities of 45.6 and 45% when samples were collected from feeding equipment or collection equipment compared with directly from the cows' teat. Collection of samples from feeding equipment was associated with a 3.40 log cfu/ml increase in CC (95% BCI: 1.26–5.59), and a tendency for increased CC was identified when samples were collected from collection equipment (1.49 log cfu/ml, 95% BCI: −0.28 to 3.03) compared with samples collected directly from the cows' teat.

The method of colostrum collection equipment cleaning was associated with CC with a stability of 19.9%. Compared with using water, the use of peracetic acid was associated with a −1.66 log cfu/ml (95% BCI: −2.73 to −0.54) reduction in CC and the use of parlor wash was associated with a 1.28 log cfu/ml (95% BCI: 0.05–2.46) increase in CC. Hypochlorite tended to decrease CC (−0.64 log cfu/ml, 95% BCI: −2.29 to 0.75) and no difference was found when soap was used (0.03 log cfu/ml, 95% BCI: −2.41 to 3.06). The cleaning of colostrum collection equipment less frequently than every use was associated with a 1.68 log cfu/ml (95% BCI: 1.19–2.18, stability 17.9%) increase in CC, and the wiping of teats prior to colostrum collection was associated with a −2.33 log cfu/ml (95% BCI: −3.46 to −1.53, stability 11.4%) reduction in CC.

**Figure 3:**
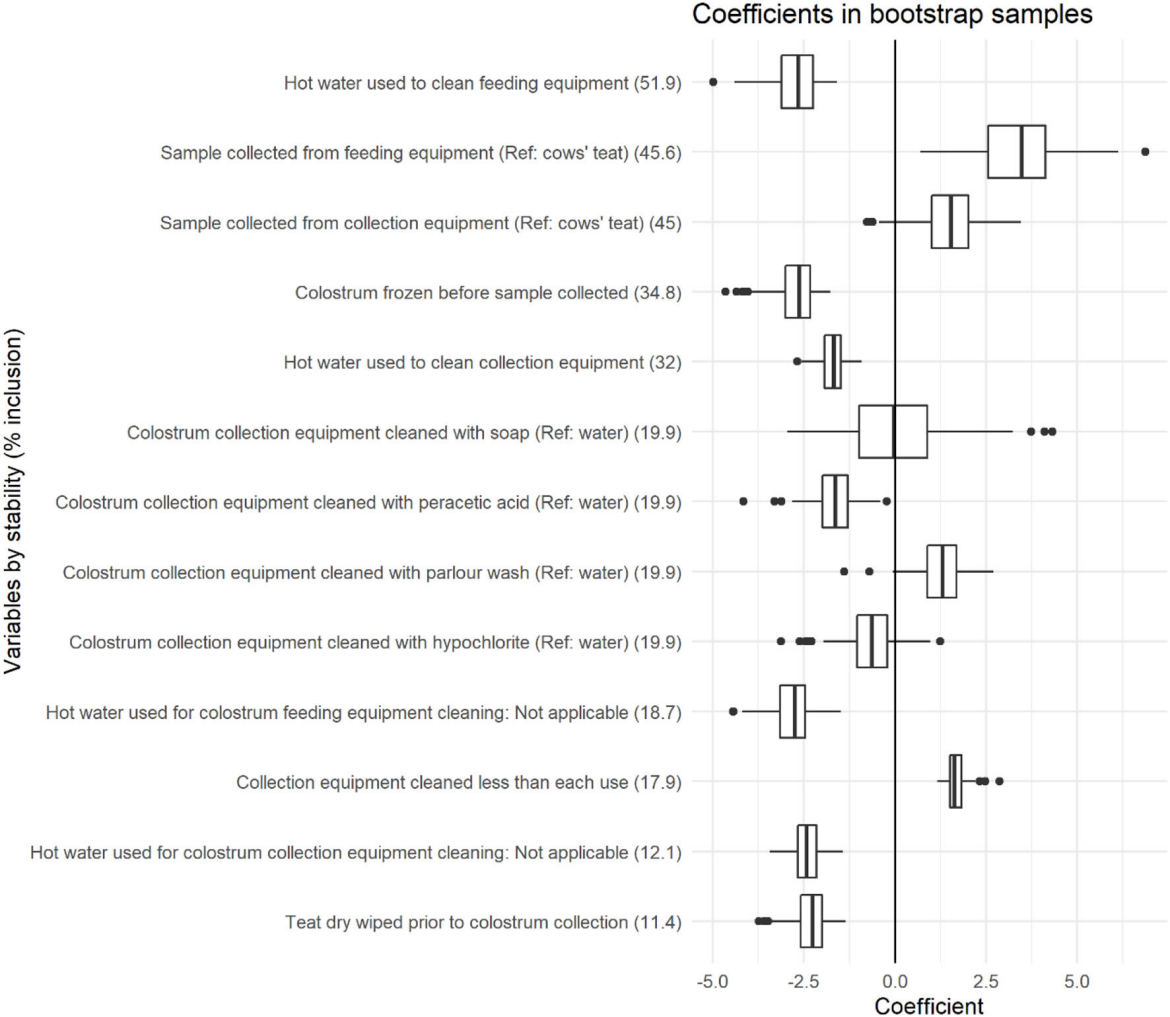
Coefficient distributions and variable stability for variables selected in at least 10% of models across 1,000 bootstrapped samples. Coefficient estimates represent the change in coliform count (log cfu/ml), and variable stability is presented within brackets for each variable.

**Figure 4:**
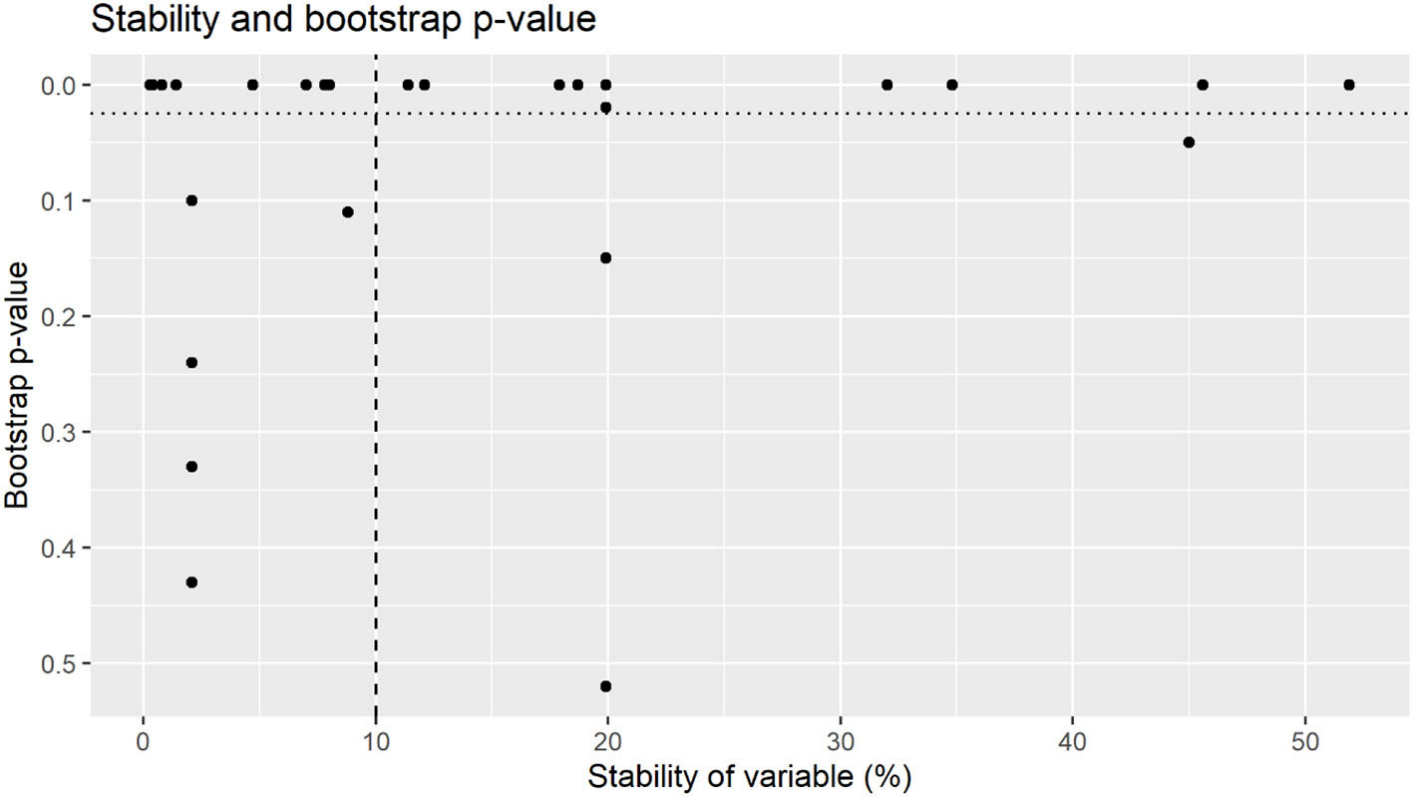
Bootstrap *p*-value by stability of variables for coliform counts. Variables were selected for final model were above 10% stability (dashed line) with a bootstrap *p*-value of <0.025 (dotted line).

Variables with >10% stability and <0.025 bootstrap *p*-value are depicted in Figure 3, with stability estimates of variables being presented in Figure 4. Coefficients, 95% BCI and stability estimates for all model variables are presented in Table 3.

**Table 3:** Stability, mean coefficient (log cfu/ml), 95% bootstrap confidence intervals and bootstrap *p*-value for all variables associated with coliform count.

**Variable**	***N***	**Stability**	**Mean coefficient**	**95% bootstrap confidence interval**	**Bootstrap *P*-value**
Hot water used to clean feeding equipment	102	51.9	−2.72	(−4.01 to −1.82)	<0.01
Sample collected from feeding equipment (ref: cows' teat)	151	45.6	3.40	(1.26 to 5.59)	<0.01
Sample collected from collection equipment (ref: cows' teat)	119	45	1.49	(−0.28 to 3.03)	0.05
Colostrum frozen prior to sample collection	14	34.8	−2.70	(−3.93 to −1.92)	<0.01
Hot water used to clean collection equipment	145	32	−1.72	(−2.35 to −1.26)	<0.01
Colostrum collection equipment cleaned with hypochlorite (ref: water)	54	19.9	−0.64	(−2.29 to 0.75)	0.15
Colostrum collection equipment cleaned with parlor wash (ref: water)	94	19.9	1.28	(0.05 to 2.46)	0.02
Colostrum collection equipment cleaned with peracetic acid (ref: water)	31	19.9	−1.66	(−2.73 to −0.54)	<0.01
Colostrum collection equipment cleaned with soap (ref: water)	12	19.9	0.03	(−2.41 to 3.06)	0.52
Hot water used for colostrum feeding equipment cleaning: not applicable	177	18.7	−2.82	(−4.15 to −2.00)	<0.01
Collection equipment cleaned less than each use	201	17.9	1.68	(1.19 to 2.18)	<0.01
Hot water used for colostrum collection equipment cleaning: not applicable	58	12.1	−2.40	(−3.32 to −1.64)	<0.01
Teat dry wiped prior to colostrum collection	270	11.4	−2.33	(−3.46 to −1.53)	<0.01
Colostrum frozen prior to sample collection: not applicable	58	9.6	−0.63	(−1.90 to 0.13)	0.10
Pre-milking teat disinfection used	271	8	1.99	(1.46 to 2.70)	<0.01
Colostrum collection equipment cleaning: not applicable	58	7.8	−1.89	(−2.82 to −0.84)	<0.01
Colostrum pasteuriser used	11	7	−3.89	(−5.36 to −3.13)	<0.01
Collection equipment cleaned less than each calf	201	4.7	−2.13	(−2.57 to −1.73)	<0.01
Colostrum feeding equipment cleaned with hypochlorite (ref: water)	45	2.1	−0.78	(−4.47 to 1.99)	0.33
Colostrum feeding equipment cleaned with parlor wash (ref: water)	24	2.1	−0.17	(−3.96 to 3.49)	0.43
Colostrum feeding equipment cleaned with peracetic acid (ref: water)	24	2.1	−0.76	(−3.85 to 0.57)	0.24
Colostrum feeding equipment cleaned with soap (ref: water)	24	2.1	3.13	(−2.72 to 5.37)	0.10
Milking system: parlor (ref: cows' teat)	220	1.4	1.75	(0.57 to 3.23)	<0.01
Milking system: robot (ref: cows' teat)	50	1.4	2.18	(0.40 to 3.97)	<0.01
Number of days between calving pen clean out	328	0.4	0.68	(0.65 to 0.74)	<0.01

N represents the number of samples where variable was “positive.”

## Discussion

This is the first study to report TBC and CC in colostrum samples from GB dairy farms and provides and initial benchmark of colostrum hygiene. Samples collected directly from cows' teats had relatively low levels of bacterial contamination with only 6.9% of samples being above threshold for either TBC or CC, compared with 37.0 and 34.4% of samples collected from collection and feeding equipment, respectively. This suggests that bacterial contamination is not likely to originate from the cow, and rather from the milking machine, collection buckets and feeding equipment as has been previously suggested (19).

Several variables were identified as having both relatively high stability and having a relatively large effect size in reducing TBC and/or CC in colostrum. The use of a milking machine to harvest colostrum was associated with increased bacterial counts, and samples from both collection and feeding equipment were associated with higher bacterial counts compared with those collected directly from the cows' teat. This large and consistent effect size for both TBC and CC suggests interventions should be targeted primarily at equipment hygiene protocols. The use of hot water was found to have a relatively large effect on bacterial counts, with samples collected from both collection equipment and feeding equipment cleaned with hot water being associated with significantly lower bacterial counts than those where cold water was used. In addition to a large effect size, these were relatively stable variables, suggesting that interventions focused on these variables would have a substantial effect on a large number of farms. As hot water was often not used to clean equipment for collection (46.3% of samples) or feeding (32.4% of samples) equipment, this represents an easy intervention for veterinarians to target on a large number of farms that could have a substantial and immediate impact on colostrum hygiene for GB dairy farms.

Disinfection of collection equipment with either hypochlorite or peracetic acid was found to have a relatively large effect size and high stability for reducing both TBC and CC. Only 31.5% of samples were collected from farms using hypochlorite of peracetic acid to clean colostrum collection equipment, with the remaining farms predominantly using either parlor washings or water. Given the large number of farms following ineffective disinfection protocols this again represents a relatively straightforward intervention to target on the majority of farms. The use of a colostrum pasteuriser was consistently associated with reduced TBC and CC, with a large effect size relative to other variables. The stability however was relatively low, at 10.9 and 7.0% for TBC and CC, respectively, suggesting that whilst colostrum pasteurization is likely to have a large effect size in reducing TBC and CC in a small number of cases, it does not seem to have a significant effect in many of the bootstrap samples taken from the original dataset. This is likely due to the infrequent use of pasteurization equipment in this sample, with only 4.1% of samples collected from farms using a pasteuriser and therefore many bootstrap samples will not contain any samples collected after pasteurization. Whilst colostrum pasteurization appears to have a relatively large effect size in reducing TBC and CC as described previously (8, 10), the relative scarcity of colostrum pasteurization and the requirement for initial financial investment may make this a more challenging intervention for veterinarians to implement on a large number of farms.

Based on the results from this trial, practical recommendations for veterinary intervention should focus on the effective cleaning of colostrum collection and feeding equipment after every use with hot water as opposed to cold water, and hypochlorite or peracetic acid as opposed to water or parlor wash. Cows' teats should be prepared with a pre-milking teat disinfectant and wiped with a clean, dry paper towel prior to colostrum collection, and colostrum should be pasteurized where possible. Only 23 samples in the current dataset were collected from farms following the optimal collection practices identified in this study (cleaning both collection and feeding equipment each time they were used with hot hypochlorite or peracetic acid and using a pre-milking teat disinfection and dry wipe prior to collection, but excluding pasteurization). By following these simple recommendations, it is likely that significant reductions in both TBC and CC will be achieved, although these results should be validated in a randomized controlled trial in future research.

Whilst some factors might have a large impact on colostrum hygiene (relatively large effect size), this might only be applicable to a small number of farms (low stability). Similarly, some factors are applicable to a large number of farms (high stability) however only have a small impact on colostrum hygiene [relatively small effect size. The recommendations from this trial are not intended to provide an exhaustive list of all factors affecting colostrum hygiene, rather identify a small number of practically implementable interventions that had the largest effect size on the majority of farms. The stability thresholds applied to select influential variables aimed to identify the most stable variables with the largest effect size (31) as shown in Figures 2, 4. Whilst there are several key variables identified in this research, it is likely that there are other variables that also impact colostrum bacterial levels to some degree Whilst the bootstrapped regression methods utilized in this research have identified several variables with both high stability and relatively large effect sizes, it is possible that variables with an effect on colostrum bacteriology levels may have remained undetected due to sample size constraints. An a priori sample size calculation was not performed, and in the absence of prior literature to base a sampling number six samples from each farm was chosen to establish a representative set of samples for each farm given financial constraints. Whilst the use of bootstrapped mixed effects models means a conventional sample size calculation is unlikely to be appropriate, the standard deviation for TBC from the current research (log 3.3 cfu/ml) and 328 samples, a conventional sample size calculation indicate an 80% chance of detecting a log 1.0 cfu/ml change in TBC. Whilst this method of sample size calculation would not be appropriate when using bootstrapped mixed effects models, it suggests that variables with relatively small effect sizes might only be detected if a larger sample size was available.

Whilst TBC and CC are highly correlated, it has been suggested that CC might be a better predictor of disease. Whilst a threshold of CC < 10,000 cfu/ml is a reasonable threshold to aim for, the negative linear relationship between CC and serum IgG suggests there is no optimal threshold, and it may be better to aim for as low as possible (10). A CC target of as low as possible would be supported by the current trial, with the 41% of samples from feeding equipment having zero CC suggesting that 0 cfu/ml is an achievable target for coliforms in colostrum from GB dairy producers.

Whilst all milking machine use was associated with higher levels of bacterial contamination, robot milking machines were associated with a particularly high level of contamination. This may be due to default settings for the collection of colostrum by the robot rather than any inherent issue with robot systems themselves. Although the default setting for robots participating in this trial for routine milk collection was generally to perform a full machine wash and perform pre-milking teat cleaning prior to milking, these were often not performed prior to the collection of first colostrum. Veterinarians on robotically milked dairy farms should investigate default colostrum collection settings and ensure that settings are configured for optimal colostrum hygiene, and future research should aim to validate how the hygiene of robotically collected colostrum might be optimized.

Farmer collection of colostrum samples represents a potential limitation of this study, as there is likely to be a degree of inconsistency in sampling technique. Variability in sample collection technique between farmers is likely to be random and is unlikely to introduce bias into models. It is possible that by being enrolled in a trial, an element of bias may have been introduced with farmers being keen to process colostrum in a relatively hygienic manner. The simple act of benchmarking has been reported to decrease levels of FPT from 21 to 11% after a benchmark report and change in management (32), although every effort was made to encourage farmers to collect samples as normal. This bias was limited by the design of the study to some degree, as farmers were only asked about collection protocols used for the samples after the samples were received. As enrolment in the trial was voluntary it is possible that farmers on this trial represent a more progressive population, and therefore estimates of bacterial contamination levels are likely to be conservative. Concurrent research was also being undertaken on the study farms which may have introduced a level of bias, particularly an intervention trial aimed at increasing growth rates in preweaned calves. One component of this was encouraging farmers to use hypochlorite or peracetic acid when collecting colostrum, however, on further analysis, only 28 samples (8.5%) were from intervention farms where farmers were now using a recommended cleaning product where they were not previously, compared with 15 samples (4.6%) from control farms. Differences between control and intervention groups were not significant after performing a chi-squared test (*p* = 0.16), and the authors feel that whilst this may have had a small effect on the numbers of farms using hypochlorite or peracetic acid overall, this is unlikely to have a significant effect on bacterial estimations and have no effect on model performance or recommendations from this trial.

Stability of variables in predicting coliform counts were far lower than TBC. Log transforming total bacterial counts resulted in a gaussian distribution for TBC. Due to a large number of zero counts, however, CC did not fit a gaussian distribution after log transformation. The distribution of residuals for CC models were carefully checked however and were deemed to provide satisfactory evidence of model fit. Furthermore, any prediction errors at extreme values are likely to be ameliorated by the bootstrapping process. The use of regularized regression models was also considered due to their effective performance in robust variable selection (33), however, due to the presence of multiple samples from each farm and relatively few explanatory variables, mixed models were better suited to the dataset. The recommendations from this research are likely to be applicable to dairy farms in GB, however caution should be taken when extrapolating the results to dairy farms in other countries.

## Conclusion

Colostrum sampled from collection or feeding equipment had higher levels of TBC and CC than those taken directly from cows' teats suggesting microbiological contamination is likely to occur from improperly cleaned equipment rather than the cow. Whilst extremely low bacterial counts were achievable, this study indicates over one third of samples collected from either collection buckets or feeding equipment were over conventional thresholds for either TBC or CC, and would, therefore, represent a significant risk for both the ingestion of pathogens and the failure of passive transfer of immunity on GB dairy farms.

Routine testing of colostrum bacteriology is relatively cheap and straightforward and is likely to be currently underutilized in the UK. Veterinarians should consider routine colostrum hygiene testing as part of a preventative calf health approach, and this trial has identified a small number of variables that are likely to have a substantial impact on colostrum hygiene for a large proportion of farms. Key recommendations based on this research to reduce bacterial levels in colostrum suggest protocols should include the cleaning of colostrum collection and feeding equipment after every use with hot water as opposed to cold water, and hypochlorite or peracetic acid as opposed to water or parlor wash. Cows' teats should be prepared with a pre-milking teat disinfectant and wiped with a clean, dry paper towel prior to colostrum collection, and colostrum should be pasteurized where possible.

## Data Availability Statement

The raw data supporting the conclusions of this article will be made available by the authors, without undue reservation.

## Ethics Statement

The animal study was reviewed and approved by University of Nottingham. Written informed consent was obtained from the owners for the participation of their animals in this study.

## Author Contributions

RH conducted farm visits and farmer training. RH, MG, CH, and PD wrote the manuscript. All authors contributed to the article and approved the submitted version.

## Conflict of Interest

The authors declare that the research was conducted in the absence of any commercial or financial relationships that could be construed as a potential conflict of interest.
